# Medical Items

**Published:** 1893-06

**Authors:** 


### *MEDICAL ITEMS.*

A LADY in distress wrote the following urgent note to her physician: Dear Dr.—Please come at once and bring your urethra with you." He took a catheter.—*New Orleans Medical and Surgical Journal.*

THE Florida State Medical Association, at its annual meeting in Jacksonville, elected the following officers: President, Dr. F. H. Caldwell, of Sanford; First Vice-President, Dr. J. A. Wakefield, of Jacksonville; Second Vice-President, Dr. R. P. Izler, of Ocala.

IN the announcement of the summer course of lectures at the Chicago Post-Graduate Medical School, Dr. Emery Lanphear is put down as a resident of New York. Dr. Lanphear is from Kansas City, and wishes the above error corrected. The subject of his lectures will be "Some Achievements in Intra-Cranial Surgery."

A NEW medical school has been chartered in Richmond, Va., to be called the College of Physicians and Surgeons. The faculty will be composed of some of the best known of the practitioners of Richmond. Dr. Hunter McGuire will be chairman of the faculty and professor of clinical surgery. From the announcement, we are encouraged to believe that the new school will be one of high grade.

THE following original lines appear on the bill heads of a Kansas physician. In admiration of the sentiment we are willing to excuse the grammar:

"If you pay your physician promptly he will attend you promptly, night or day, rain or shine, while your slow neighbor suffers and waits, as he made the doctor wait, and while he is waiting the angels gather him in."

DR. CHARLES CARROLL LEE, of New York, died on the 10th of May. Dr. Lee was one of the most prominent of the New York physicians, and his death at the height of his fame and usefulness will be a loss to the profession of that city. At the time of his death he was professor of gynecology in the New York Post-Graduate Medical School, consulting surgeon to several hospitals and a member of many important medical societies.

---

IT will be remembered by our readers that sometime ago Dr. R. G. Eccles published in the *Druggists' Circular* the result of his analysis of Radam's Microbe Killer, stating that it contained sulphuric, sulphurous, and hydrochloric acids, together with some wine and water. In a paper published in the West, Mr. Radam denied the correctness of Dr. Eccles' analysis, and spoke of the Doctor as a quack and a charlatan. For this libel Dr. Eccles commenced suit and has been awarded damages to the amount of $6,000.—*Brooklyn Medical Journal.*

---

FROM a paper by Dr. Billings in the June *Forum* it appears that France is not the only country in the world in which the birth-rate is on the decrease in proportion to the population. The birth-rate is diminishing all over the United States, with the exception of three or four States and Territories in the untutored West. The greatest decrease has occurred in the South, and the least in the Northern States. About two years ago a prize was offered in France to the town which would show within a certain time the highest birth-rate. Such an offer in France we can imagine was followed by lively competition. Will not some philanthropic citizen of the fashionable one-offspring aristocracy do something to stimulate our own infant industries? Fifth avenue, New York, and Prairie avenue, Chicago, to the rescue!

